# COVID-19 pandemic: do we need systematic screening of patients with cardiovascular risk factors in Low and Middle-Income Countries (LMICs) for preventing death?

**DOI:** 10.11604/pamj.2020.35.2.22947

**Published:** 2020-04-29

**Authors:** Mazou Ngou Temgoua, Liliane Mfeukeu Kuate, William Ngatchou, Aurelie Sibetcheu, Zouliatou Nzina Toupendi, Grace Belobo, Alice Ossa, Samuel Kingue

**Affiliations:** 1 Faculty of Medicine and Biomedical Sciences, Department of Medicine and Specialities, Yaoundé, Cameroon; 2 Faculty of Medicine and Pharmaceutical Sciences, Department of Surgery, Douala, Cameroon; 3 Faculty of Medicine and Biomedical Sciences, Department of Pediatrics, Yaoundé, Cameroon; 4 Faculty of Medicine and Biomedical Sciences, Department of Radiology and Medical Imaging

**Keywords:** COVID-19, low and middle income countries, cardiovascular risk factors

## Abstract

COVID-19 pandemic is an emergent cardiovascular risk factor and a major cause of mortality worldwide. Thromboembolism is highly suspected as a leading cause of death in these patients through vascular inflammation caused by SARS COV2. Until now there is no real treatment of COVID-19 and many proposed drugs are under clinical trials. Considering the high incidence of thromboembolic events in critically ill patients with COVID-19, prevention of this disorder should be essential in order to reduce mortality in these patients.

## To the editors of Pan African Medical Journal

COVID-19 pandemic is a global public health issue; it´s caused by SARS Cov 2 (severe acute respiratory syndrome coronavirus 2) a seventh coronavirus which causes the disease in humans. The first cases were discovered in Wuhan in China and is being exported to a growing number of countries. By March 16, 2020, World Health Organization (WHO) reported that 143 countries were affected with 153 517 cases and 5735 deaths [1]. The high level of mortality has lead all countries and all non-governmental organizations to pay particular attention to this emergency in order to curb the burden of the disease [2]. This infection is primarily spread from human to human via droplets during close unprotected contact with infected people. The virus invades the alveoli and links to the Angiotensin Converting Enzyme 2 (ACE2) receptor of type 2 pneumocytes by their Spike protein. Clinical manifestations of the disease are related to both direct effects of the virus or inflammatory mediators (IL1, IL6 and TNF alpha); the symptoms include fever (88.7%), cough (67.8%), fatigue (38.1%), sputum production (33.4%), shortness of breath (18.6%), sore throat (13.9%), and headache (13.6%). In addition, some patients have gastrointestinal symptoms, with diarrhea (3.8%) and vomiting (5%). Major complications of the disease include: Acute respiratory distress syndrome, septic shock, multivisceral distress syndrome and death [3,4]. Several studies have shown that patients who are at increased risk of death are those with cardiovascular risk factors like: age >60years, obesity, hypertension, diabetes and people with history of chronic respiratory disease, pregnant women and cancer [3-5].

Cardiovascular risk factors increase death by several mechanisms during COVID-19 infection. In obese patients there is an increased risk of inflammation due to adipose tissue potential. Hyper expression of ACE2 are also found in obese patients and thromboembolism events are frequent [6]. SARS Cov2 induces an alteration of insulin secretion and may increase resistance to insulin action, this could lead to acute decompensation of diabetes [7]. A high level of troponin and natriuretic peptides have been found in critically ill patients, the main proposed mechanism is inflammation of the vascular system than can result in diffuse microangiopathy with thrombosis. Inflammation of the myocardium can induce myocarditis, heart failure, cardiac arrhythmias, acute coronary syndrome, rapid deterioration and sudden death [8]. Worldwide strategy to diagnose the disease is through direct identification of the virus by RT-PCR and/or Thoracic CT-Scan showing ground-glass opacity (56.4%) and bilateral patchy shadowing (51.8%) [4]; apart from these gold standards, rapid diagnostic tests have been proposed for systematic screening and validation is currently done in several countries [4]. In Low and Middle-Income Countries (LMICs), like all over the world, there is an increased spread of the COVID-19 [9] and high burden of cardiovascular diseases [10]; but until now there is no political strategy to screen systematically patients with cardiovascular risk factors for early management. We think that systematic screening of COVID-19 in patient with cardiovascular risk factors or established cardiovascular diseases could help to reduce the burden of the disease in this continent. This strategy could be particularly important because in most of these countries there is lack of adequate resources to support patient in critical conditions.

## Conclusion

In LMICs there is an urgent need to target patients with cardiovascular risk factors or established cardiovascular diseases for earlier screening of COVID-19 and better managment of these patients in order to prevent death linked to the virus.

## Competing interests

The authors declare no competing interests.
